# Do storage solutions protect endothelial function of arterialized vein graft in an experimental rat model?

**DOI:** 10.1186/s13019-020-1077-6

**Published:** 2020-02-10

**Authors:** Olivier Fouquet, Jean-David Blossier, Simon Dang Van, Pauline Robert, Agnès Barbelivien, Frédéric Pinaud, Patrice Binuani, Maroua Eid, Daniel Henrion, Christophe Baufreton, Laurent Loufrani

**Affiliations:** 1 Department of Cardiac Surgery, University Hospital of Angers, France, 4 rue Larrey, 49933 CHU Angers Cedex 9, France; 2 Institute MITOVASC CNRS UMR 6015, INSERM 1083, Angers, France; 3 Department of Cardiac Surgery, CHU Dupuytren, Limoges, France; 4 University Hospital of Angers, Angers, France

**Keywords:** Coronary artery bypass grafting, Venous graft, Storage solutions, Vascular reactivity

## Abstract

**Background:**

This study aims to compare the effects of storage solutions commonly used in coronary artery bypass grafting on the vascular reactivity in vein graft interposed in arterial position in syngeneic rats.

**Methods:**

Twenty-seven male Lewis rats were sacrified to sample a vein graft implanted 6 weeks ago into abdominal aorta position. The vein grafts were inferior venae cavae initially pretreated with heparinized saline solution (HS) or autologous heparinized blood (AHB) or our referent solution, GALA. The endothelial functionality, the in situ Reactive Oxygen Species (ROS) levels and the histological characteristics were conducted from segments of arterialized vein graft.

**Results:**

At 6 weeks, graft thrombosis occurred respectively in 22% of AHB group, 62.5% in the HS group and 82.5% in the GALA group. In each group, significative intimal hyperplasia was observed. After 6 weeks, an endothelium-remodeling layer associated with an increase of wall thickness was observed in each group. Endothelium-dependent tone was reduced in the vein graft regardless of the group. No difference was observed concerning the ROS in vein graft between the different groups. In distal aortic sections, ROS levels were increased in HS and GALA groups.

**Conclusions:**

Storage solutions used in this experimental model of vein graft implanted in arterial position cause graft injury and a complete disappearance of vascular reactivity. GALA solution did not reduce intimal risk hyperplasia when the vein graft was exposed to arterial flow in a rat model.

## Background

Ischemic heart disease remains the leading cause of death [1] in developed countries and higher life expectancy increases its incidence. Coronary artery bypass grafting (CABG) is considered the best treatment in most cases [2]. The success of surgical approach depends on the long-term patency rate of the grafts used. Saphenous vein graft remains the most frequently conduit used in CABG but early occlusion rates were reported ranged from 15 to 26% at 1 year resulting of vein graft dysfunction [3]. The principle etiologies of early postoperative graft failure are technical operative errors, low flow, thrombosis and vasospasm. At mild term a graft failure can appear, and it is histologically characterized by an intimal hyperplasia (IH) which is a proliferation of smooth muscle cells (SMC) and a deposition of extracellular matrix (ECM) in the intima. An atherosclerosis mechanism is observed with a risk of vein graft thrombosis [4, 5].

To preserve the SVG quality as well as endothelial cells (EC) viability and functionality, some intraoperative graft storage solutions have been evaluated in-vitro [6, 7] and some were developed for vascular conduit preservation [8, 9]. The GALA solution (Glutathione, L-ascorbique acid, L-arginine and glucose; pH = 7.4), being widely recognized in this purpose; its effects compared to other solutions (saline and autologous blood with added heparin) maintained the endothelial function and structural viability of the grafts for 1 to 24 h of storage [10]. A sub study of the PREVENT IV randomized trial demonstrated that buffered saline solution had lower vein graft failure rate studied by angiography 12 to 18 months after surgery and better clinical outcomes compared to saline or autologous blood solutions [11].

When venous graft is exposed to the arterial blood flow, hemodynamic strengths (shear stress (SS) and cyclic strain (CS)) are applied on the vascular wall. The impact of SS on EC depends on the type flow applied on the venous graft [12]. IH occurs in approximately 50% of SVG and is characterized by SMC migration from the media to the intima through metalloproteinase (MMPs) activation [13]. Vein graft failure response to hypoxia-reoxygenation injury associates mechanisms of vasospasm, adhesion cascade, ROS (Reactive Oxygen Species) production, platelet aggregation, endothelial cells loss, and shear stress stimulation during the first week [14].

The research concerning storage solutions is in vitro human studies and it is clearly accepted that they have a fundamental role in the early preservation of the endothelium of the vein graft [15]. However, when the venous graft was exposed to arterial shear-stress and cyclic strain, smooth muscle cells change their phenotype from a contractile to a secretory stage [16]. Physiologically, venous endothelium produces small amount of NO while in arterial position, the production decreases. The storage solution should maintain a physiological pH and support endothelial NO production by provision of an eNOS substrate [17]. A lot of intraoperative vein graft preservation solutions were studied but we focused three of them according to our clinical practice and following the results of experimental study [10]. The purpose of this study was to evaluate if autologous heparinized blood, heparinized saline serum and GALA considered as our referent solution could have a protective effect on an arterialized vein graft in a rat model.

## Methods

**Fig. 1:**
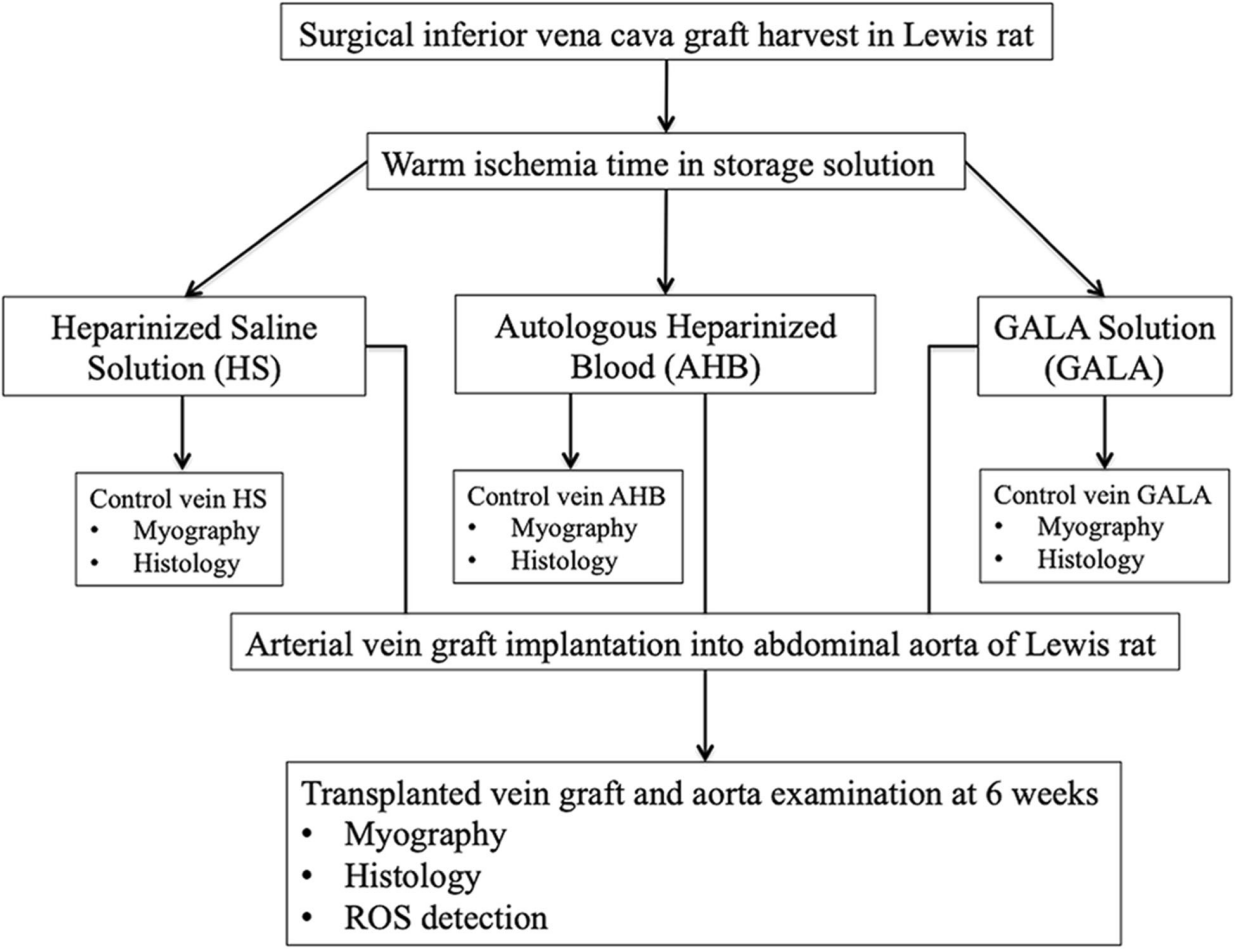
Study flow-chart

Figure 1 shows the flowchart of the different steps.

### Preservation solution

The inferior venae cava grafts were randomly stored in the different preservation solutions at room temperature (20 °C) before implantation. Three different storage solutions were used for the ex-vivo preservation of explanted veins. Vein grafts were stored in one of the following solutions: autologous heparinized (40 UI/ml heparin choay) blood (AHB), 0.9% sodium chloride heparinized (40 UI/ml heparin choay) serum (HS); GALA solution (gluthion, ascorbic acid, L-arginine) prepared at the hospital pharmacy which constitutes in our institution the referent storage solution used in CABG for vein grafts. GALA solution was composed of physiologic salt solution to which was added: 0.02 ml magnesium sulfate, 1.785 mg potassium hydrogenophosphate, 0.03 ml magnesium chloride, 0.042 ml calcium chloride, 0.08 ml potassium chloride, 5000 UI/ml heparin choay, L-ascorbic acid (1 g/500 ml), 0.6 ml glucose 5%, 0.75 ml sodium bicarbonate, 1.2 ml sodium chloride, 1.5 ml L-arginine as a substrate for endothelial nitric oxide synthetase (eNOS) and 2.7 ml glutathione. The inferior vena cava segments were collected from 27 donor rats.

### Animals

Inbred male LEWIS (Albino rat, a/a, B/B, Tyr^c^/Tyr^c^, h/h – MHC: RT1’) rats weighting 337 ± 17 g and aged 12.8 ± 0.75 weeks (JANVIER LABS, Le Genest Saint Isle, France) were housed in a regulatory pet. No exogenous immunosuppressive drug therapy was employed. All procedures were performed according to the guidelines of the Institutional Animal Care and approved by the Ministère Français de l’enseignement Supérieur et de la Recherche (authorization number December 2014: 000375.01).

### Surgical harvest and implantation of the vein graft

Twenty-seven donor rats were anaesthetized (isoflurane 5%) and pretreated with buprenorphine (Temgesic; 0.1 ml/100 g S.C). A large incision through the abdominal wall allowed to expose the inferior vena cava and the abdominal aorta. The different branches of the vein were ligatured with 8–0 monofilament Prolene®. The donor rats were sacrificed in a CO2 chamber. The inferior vena cava was stored in a preservation solution (9 in AHB, 9 in HS and 9 in GALA) previously described. Each vein was carefully flushed with the storage solution. After storage in one of the preservation solutions and before arterial implantation, a segment of the vena cava was sampled then stored in PSS for myography and corresponding to the control vein group (AHB control vein, HS control vein and GALA control vein).

**Fig. 2:**
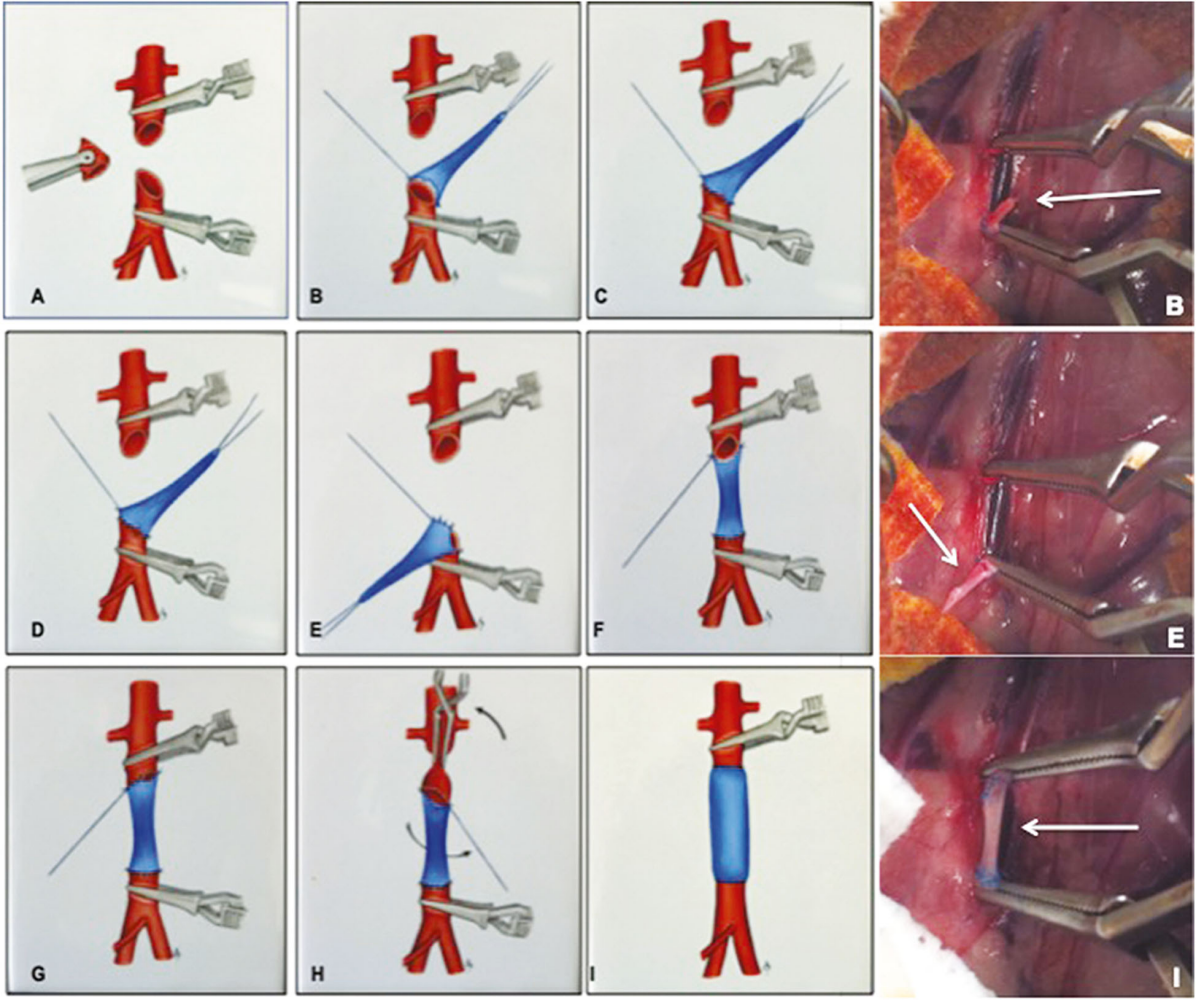
After clamping the abdominal aorta below renal arteries, an arterial segment was removed (**a**). The vein graft was interposed into the recipient aorta starting with the distal suture (**b**). The first interrupted suture concerned the posterior wall of the graft (**c**) then the anterior wall was better exposed (**d**). Pulling down from the graft exposed the posterior suture (**e**). The proximal anastomosis was performed with posterior interrupted sututre (**f**) then the anterior suture (**g**). Torsion of the graft was performed to expose the posterior wall and to finalize the suture (**h**). The clamps were removed and the patency of the graft was confirmed by recoloring the lower extremities (**i**)

Twenty-seven recipient rats were anaesthetized and pretreated as described. The abdominal aorta was exposed, isolated from the adjacent inferior vena cava and controlled with a loop between the renal arteries and the aorta bifurcation. The aorta was clamped, and an arterial segment was removed. Heparin injection (300 UI/kg) was performed during the procedure. The vein graft was interposed into the recipient’s abdominal aorta, an end-to-end anastomosis was then established between the donor and the recipient vessels, respectively, using a running 9–0 monofilament suture Prolene® at × 10 magnification (Fig. 2). At the end of this procedure, the clamps were removed, the patency of transplanted graft was confirmed and blood supply to the pelvis and lower extremities was restored. Rats were placed in an incubator at 29 °C for 20 min until fully awake and monitored for signs of lower limb ischemia, hemorrhage and pain. Antibiotics were administered for 7 days (Bactrim 2.5 ml per liter of drinking water). Buprenorphine from 0.067 to 0.33 ml per 100 g was administered in case of mild, moderate or severe pain (pain scale in rats according to the guidelines of the Institutional Animal Care) The same cardiac surgeon performed all procedures.

### Transplanted vein graft examination

At 6 weeks after transplantation, under anesthesia, the abdomen was exposed, previously implanted vein grafts with a proximal and distal segments of the aorta were removed and subsequently divided into five segments of commensurate length: two segments (proximal aorta and vein graft) were stored in a 10 ml organ bath containing a physiologic salt solution (PSS) for myography and three segments (proximal aorta, distal aorta and vein graft) were cooled in liquid nitrogen and stored at − 80 °C for histology and immunohistochemistry.

### Vascular reactivity

Myography: for each sacrificed recipient rat, 3 fresh segments of distal aorta, vein graft and control vein stored in PSS were analyzed. At day+ 1, the fresh segments were mounted on a wire-myograph (DMT, Aarhens, DK) as previously described [18]. Briefly, 2 tungsten wires (25 μm diameter) were inserted in the lumen of the grafts and connected to a force transducer and a micrometer, respectively. Vascular grafts were bathed in the PSS described above. A wall tension, equivalent to the intra-arterial tension (20 mN for aortic segment and 5 mN for control vein and vein graft segments), was applied corresponding or equivalent to arterial blood pressure at 90 mmHg. Vessels were allowed to stabilize for 20 min. Contractility was assessed with phenylephrine (PE 10–6 M). Actylcholine (Ach 10–6 M) induced relaxation was then obtained after phenylephrine-induced preconstriction (50% of maximal contraction). Vascular response to PE (from 10 to 9 M to 10–5 M), Ach (from 10 to 9 M to 10–5 M) and SNP (Sodium Nitroprusside) (from 10 to 9 M to 10–5 M) were studied.

### Histology

Vascular segments were embedded in paraffin and cooled at − 80 °C. Sections (7 μm thickness) were obtained from the fixed arterial segments and stained with orcein to visualize elastic fibers. External diameter, lumen diameter, and media thickness were determined after images acquisition (Olympus T100 microscope, Sony Camera) and analyzed using the Histolab Software (Microvision, Paris, France) for cross-sectional area (CSA) calculation as previously described [19].

### Detection of reactive oxygen species detection using confocal microscopy

Dihydroethidium staining (DHE, Sigma-Aldrich) was used to evaluate the in-situ levels of superoxide anions (O_2_^−^) [20]. DHE penetrates cells and is oxidized by superoxide (O_2_^−^) into fluorescent products that are trapped by intercalation into the DNA. Sections were incubated with DHE (1 μmol/L) in phosphate-buffered solution (PBS) and DAPI (4′,6′-diamidino-2-phénulindole-Molecular probes, Invitrogen) for nuclear cells at 37 °C for 30 min in a humidified chamber protected from light. Fluorescent images of ethidium bromide were obtained using a confocal microscope (Nikon) and quantified with the ImageJ (NIH) software. The different studied sections were vein graft and distal aorta samples.

### Statistical analysis

Data were reported as mean ± standard error the mean unless indicated otherwise. Considering the sample size, non-parametric tests (t-test or Mann-Whitney test) were conducted to assess the statistically significant of each experiment using Graphpad Prism software (La Jolla, Calif). *P* value of ≤ .05 was considered statically significant.

*Sample size*: based on a previous study [21], and to show a difference between the three groups at a power of 90% with a significance level of 5% concerning vascular reactivity and inflammatory response, a sample size of 27 rats was required for this experimental study (9 per group).

## Results

### Surgery and occlusion rate of the venous graft

The mean duration of venous storage and duration of surgery, the weight gain during the follow-up were presented in the Table 1. At 6 weeks, the mortality was 0/9 in the HS group, 1/9 in the AHB group (bleeding, *n* = 1) and 3/9 in the GALA group (bleeding, *n* = 2; mesenteric ischemia, n = 1).

**Table 1:** Surgical data

	Heparinized Saline solution HS (*n* = 9)	Autologous Heparinized Blood solution AHB (*n* = 9)	GALA solution (*n* = 9)
Duration of venous storage (min)	91 ± 56	106 ± 57	88 ± 57
Duration of venous transplantation (min)	67 ± 11	63 ± 10	67 ± 11
Weight gain during follow-up (gr)	73 ± 24	79 ± 22	69 ± 21

**Fig. 3:**
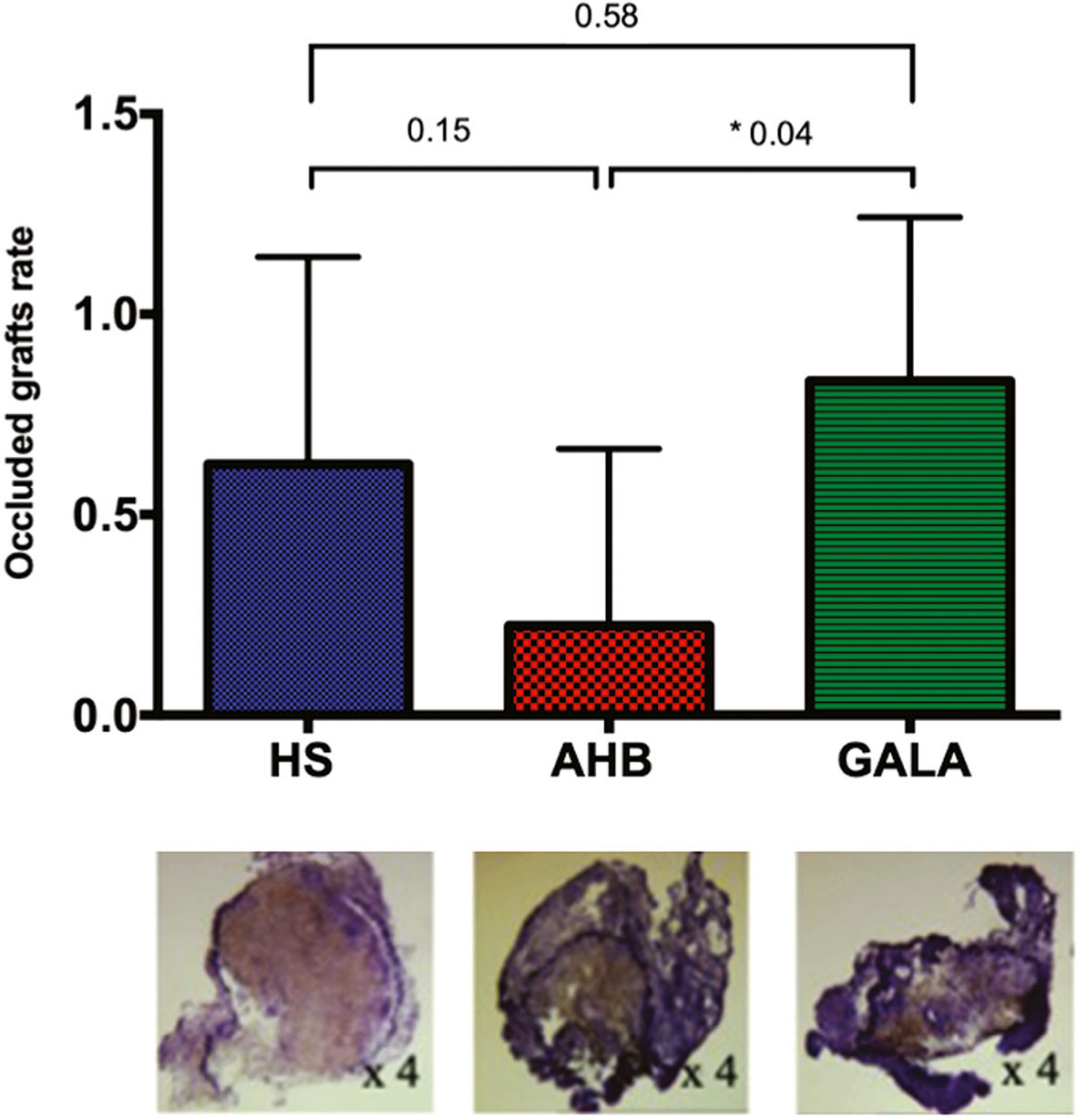
A non-parametric test was used to compare the different groups. Occluded grafts rate was higher in the GALA group than HS group (*p* = 0.04) and AHB group (*p* = 0.58)

Among rats alive, thrombosis occurred respectively in 2/9 (22%) grafts of AHB group, 5/8 (62.5%) in the HS group and 5/6 (83.3%) in the GALA group (Fig. 3). The suture analysis showed no proximal nor distal anastomoses stenosis.

### Histology

**Fig. 4:**
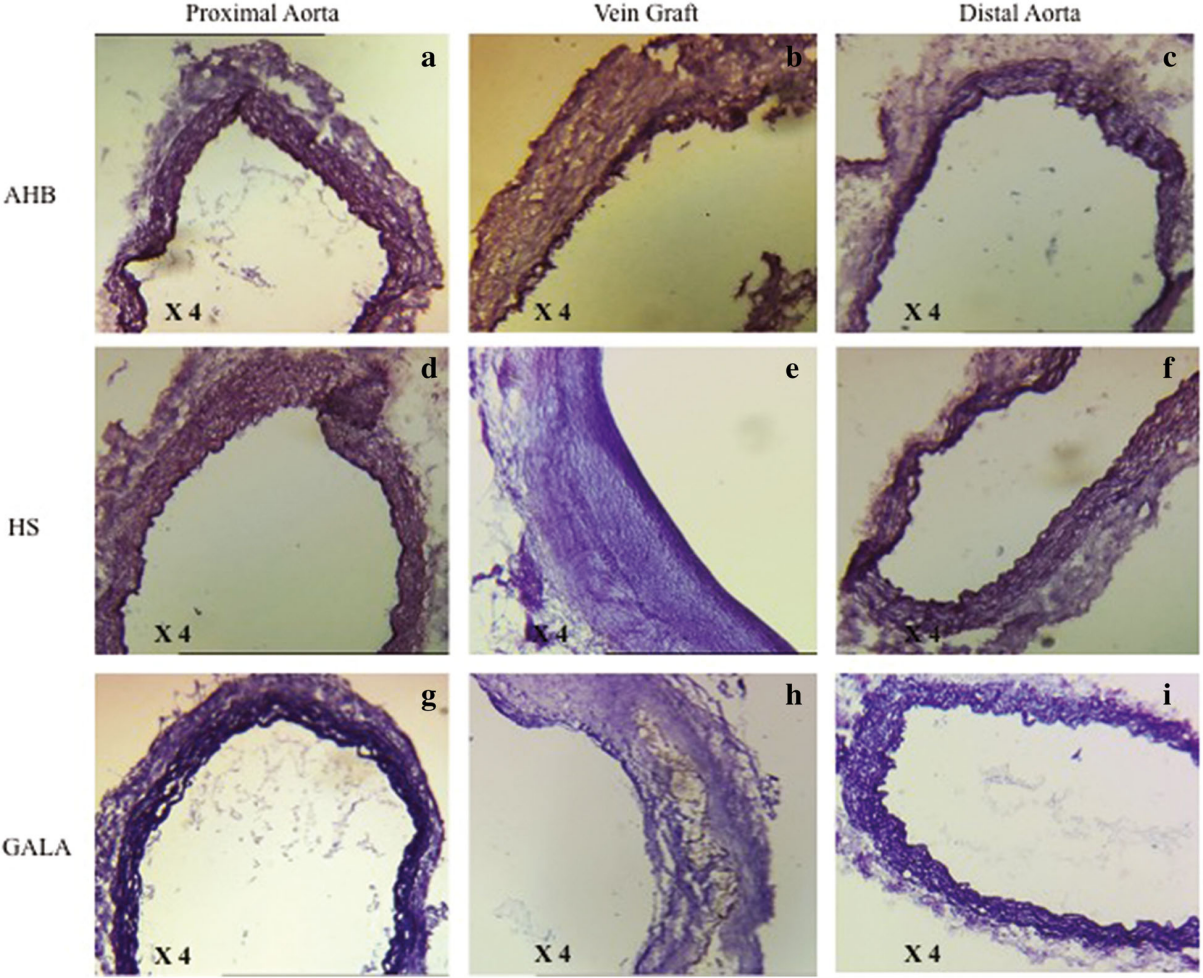
Results of arterial blood exposition for 6 weeks (orcein staining). The proximal (**a**, **d**, **g**) and the distal aorta (**c**, **f**, **i**) retain its their histological architecture. An important intimal hyperplasia was observed in the vein graft (**b**, **e**, **h**) with a disappearance of endothelium layer and a structural media failure regardless the preservation solution group

The wall thickness of control vein was 17.75 ± 3 μm in the HS group, 16.3 ± 4 μm in the AHB group and 17.3 ± 5 μm in the GALA group (0.7). The wall thickness of vein grafts was 105 ± 32 μm (+ 83.1% compared with wall thickness of control vein) in the HS group, 101 ± 25 μm (+ 83.9%) in the AHB group and 107 ± 31 μm (+ 83.8%) in the GALA group (0.8). The orcein staining showed an important intimal hyperplasia in the vein graft with a disappearance of endothelium layer and a structural media failure regardless the preservation solution used (Fig. 4). The distal aorta retains its histological architecture in each group. An intraluminal fibrosis was observed in the occluded grafts (Fig. 3).

### Myography

Because of the small number of permeable vein grafts at 6 weeks, the analysis concerned the average of the three preservation solutions groups.

Phenylephrine induced a significant contraction in the aorta (3.10^− 8^ μmol/L), about 50% in the control vein (from 10^− 6^ to 3.10^− 6^ μmol/L). No contraction was observed on the vein graft except for high doses of phenylephrine (from 10^− 6^ to 10^− 5^ μmol/L).

**Fig. 5:**
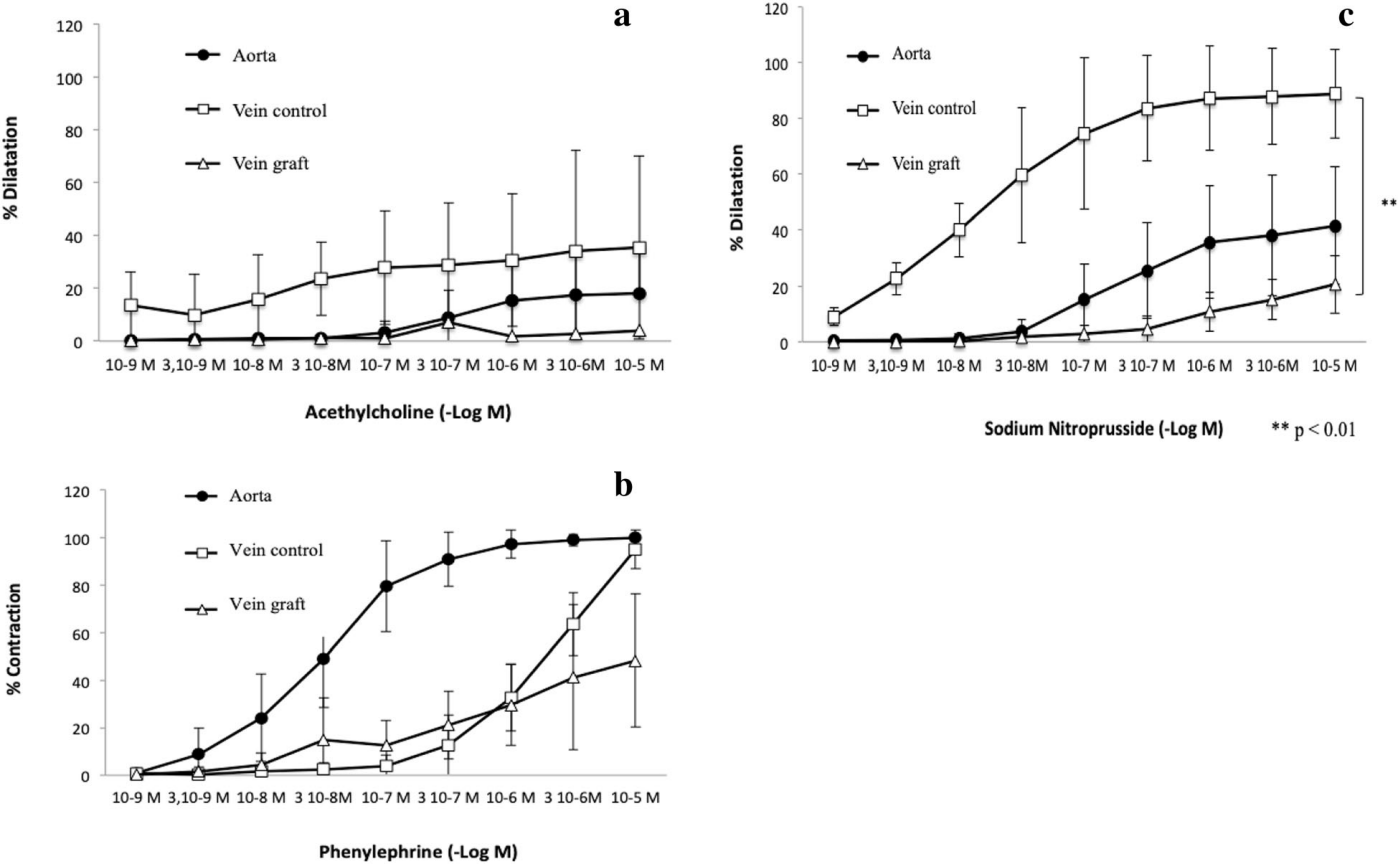
Dilatation induced by increasing dose of Acethylcholine (**a**) in phenylephrine preconstricted vein (**b**) was not observed in the different vessels. Dilatation induced by increasing dose of sodium nitroprusside was maintened in the control vein compared to the vein graft group (**c**). Vein graft = AHB vein graft (*n* = 3)+ HS vein graft (*n* = 7) + GALA vein graft (*n* = 1), *n* = 11; Control vein = AHB control vein (*n* = 9)+ HS control vein (*n* = 9) + GALA control vein (*n* = 9), *n* = 27; Aorta = AHB distal aorta (*n* = 9)+ HS distal aorta (*n* = 8)+ GALA distal aorta (*n* = 6), *n* = 23. Data are presented as mean ± SEM Double asterisk indicates *P* < 0.01 for vein control vs vein graft

Acetylcholine did not induce dilatation neither in the control vein nor the aorta and the vein graft. Sodium nitroprusside induced a significant dilatation in the control vein compared to the vein graft (*p* < 0.01) (Fig. 5). A dilatation was observed in the aorta for high dose of sodium nitroprusside (from 10^− 7^ to 10^− 5^ μmol/L).

### ROS detection

**Fig. 6:**
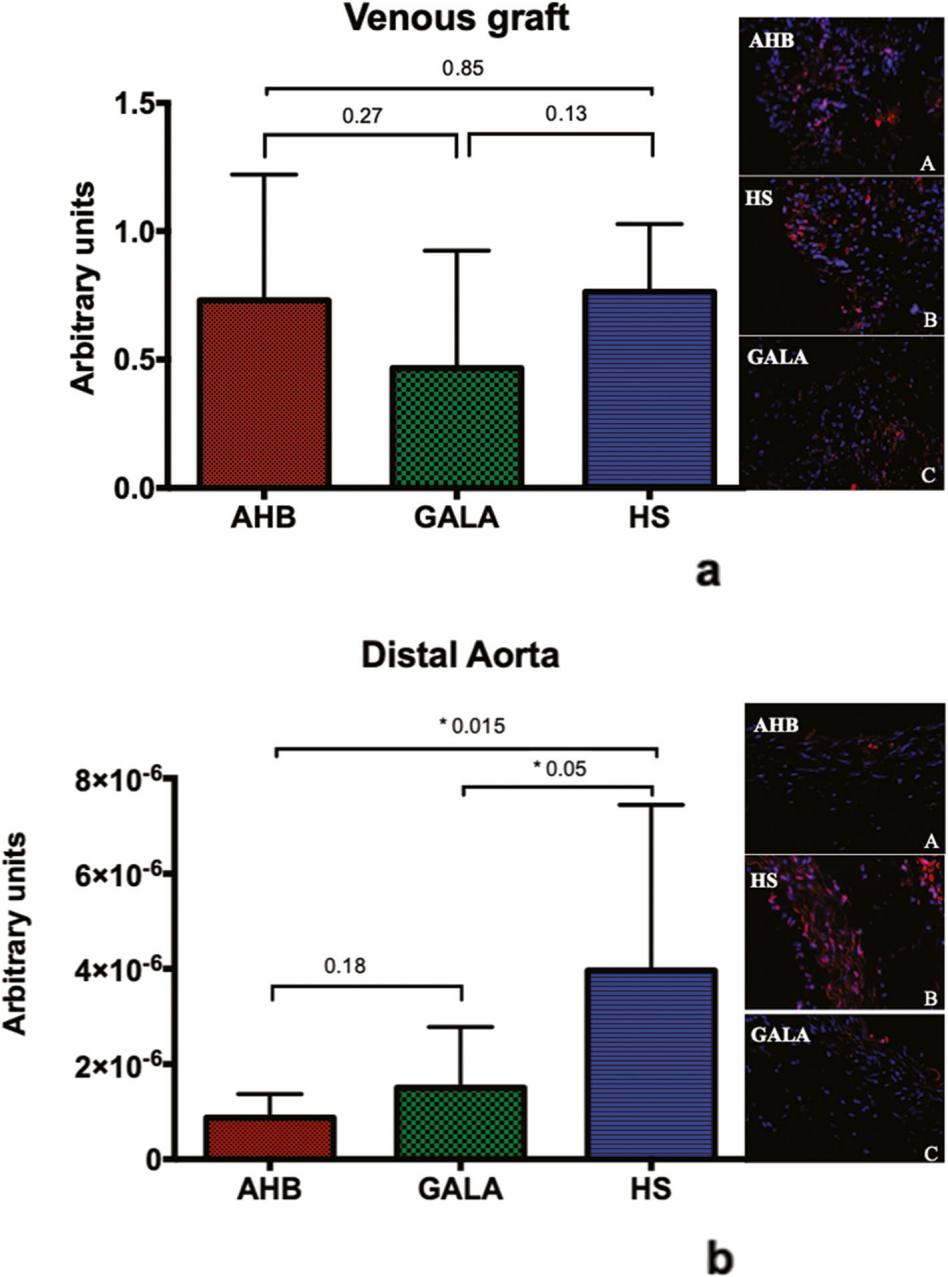
Superoxide production in the different groups is shown in the bar graphs for venous grafts section (**a**) and for distal aorta sections (**b**). Fluorescent images of ethidium bromide (DHE) are colored in red and DAPI in blue

In venous graft sections, there was no difference concerning the superoxide formation evaluated by ethidium bromide-enhanced fluorescence (Fig. 6a). Moreover, in distal aorta sections, superoxide anion levels were significantly higher in HS group than AHB and GALA groups (Fig. 6b).

## Discussion

Vein graft failure is the result of a complex mechanism associating endothelial injury which can occur from the surgical harvesting, intimal hyperplasia and atherosclerosis [22]. The main role of storage solutions is to preserve the endothelium of graft conduit and their actions run from storage period to arterial implantation [15].

### Vein graft analysis at 6 weeks

- Saphenous vein graft (SVG) represents an autologous transplantation, as it is explanted, preserved in storage solution into a cup at room temperature and then implanted in arterial system. In clinical practice, the duration of vein graft storage corresponding to the warm ischemia tissue was difficult to evaluate because no data are published. Some surgeons do not store SVG before creating the anastomoses and develop the concept of “no-touch technique” with excellent graft patency [23]. In contrast to what is done for solid organs destined for allotransplantation, where different preservation solutions were developed such as University of Wisconsin solution for pancreas or Celsior solution for heart, the normal saline solution was the first storage solution for SVG. O’Connel et al. demonstrated that 2 h of saline infusion produced an IH [24]. Saline solution has negative effects on the endothelial layers and therefore may compromise graft patency [25]. However, different in-vitro studies cannot conclude on the superiority of autologous whole blood, alternatives solutions such as storage solutions like Tiprotec™ or Somaluthion™ remain the subject of further clinical trial [25]. Other study showed that autologous whole blood had some clear advantages compared to saline solution, this difference does not persist post-arterialization [26]. In the present study, the duration of vein storage in the different solution was high as to allow preparation of the recipient rat following vein graft harvesting but no relation was found between storage duration and importance of IH. Thatte et al. showed that duration of storage time in GALA solution (from 60 min to 1440 min) did not alter smooth muscle or endothelial cell function [10]. None of the storage solutions used in this study has reduced the intimal hyperplasia. In fact, we observed a significant increase of the wall thickness and histological signs of fibrosis.

- Among the occluded veins, only an important IH could explain the vein graft occlusion. In order to obtain an arterialized vein graft, we analyzed the conduit at 6 weeks when the IH started its process. Similar results were observed in the study published by Wong et al. who performed arteriovenous fistula in a murine model and then collected for analysis at 7, 14 and 28 days postoperatively [27]. They found significant changes in the intima at 7 days, a significant hyperplasia was observed at 14 days and the patency rates at 28 days were 50%. Sun et al. investigated the efficacy of oral administration of hydrogen-rich water (HW) for prevention of intimal hyperplasia concerning inferior vena cava placed as an interposition graft in the abdominal aorta. Six weeks after bypass procedure, all vein grafts presented SMC and collagen deposits, macrophage infiltration but significantly less in the rats that consumed HW [28]. In our study, the rate of occluded vein grafts among them was greater in the GALA group than AHB and saline solution groups. GALA solution is based on a physiological salt solution and contains glutathione and L-ascorbic acid, antioxidant and arginine, a substrate for NOS (Nitric Oxide Synthetase) in EC to protect the endothelium against ischemic injury during storage. The analysis of GALA effects on EC showed a protecting endothelial structure and function [10]. According to our results, we cannot conclude that GALA solution protects the endothelial layer when the vein graft was arterialized in this rat model. These results should be interpreted with caution if we extrapolated them in human. The occluded vein graft rate at 6 weeks is low after coronary bypass but probably storage solutions did not limit vascular remodeling during the chronic intimal hyperplasia (stage III), their benefits intervening during the first weeks. Haime M et al. compared the impact of intraoperative preservation of SVG in GALA solution (Duragraft®) versus heparinized saline on vein-graft failure related outcomes after CABG in 2436 consecutive patients. In this retrospective study, the intraoperative treatment of SVGs with Duragraft was associated with a lower risk of nonfatal myocardial infarction, revascularization [29]. However, these encouraging results must be interpreted with caution and only a well-constructed randomized study will answer the real question “which solution best preserves endothelial integrity from the vein harvesting to the arterial implantation” [17].

Many studies showed that intimal hyperplasia is an adaptive mechanism following arterialization of the vein and is unlikely linked to vein graft thrombosis [30]. Arterialization of a vein appears when the graft is exposed to arterial flow. Contractile SMC become secretory and proliferative [16]. EC cannot produce NO and endothelial layer damage produces growth factors and proinflammatory cytokines (IL-6, IL-8) [31]. This process promotes leukocytes recruitment and thrombin formation [32].

- In the present study, regardless of the storage solution, no endothelium-dependent dilatation was observed when the vein grafts were mounted on a wire-myograph attesting to endothelial injury. The same findings were showed in the control vein and aorta groups. However, the SMC layer viability was observed in the control vein group, the preservation solutions maintained the SMC functionality from 88 to 106 min of storage.

The implication of the shear stress in vascular biology and inflammation was described by Touys et al. [33]. All vascular cell types produce reactive oxygen species (ROS) that regulate vascular function by modulating cell growth, apoptosis, migration, inflammation and extracellular matrix protein production. Oxidative stress and associated oxidative damage are mediators of vascular injury and inflammation and constitute the first steps of atherosclerosis development [13]. In our study, we observed an important oxidative stress in the different vein graft segments analyzed. The inflammatory response into the vein wall due to shear stress was also observed in arterial samples explaining the vascular function damage. These results should be interpreted with caution because of the small number of permeable vessels at 6 weeks. In the presence of high levels of sodium nitroprusside, a dilatation was observed in the control vein group. Saad Enouri et al. studied characteristics of myogenic reactivity in isolated rat mesenteric veins. The integrity of the endothelium was assessed by a dilator response to acetylcholine in phenylephrine-preconstricted veins. The veins were able to develop significant myogenic tone that appears greater over the low-to-intermediate pressure ranges, but mesenteric veins did not demonstrate myogenic responses unlike mesenteric arteries [34]. In our study, the control vein presented an endothelium-dependent relaxation injury with a preservation of smooth muscle cell (SMC) activity (endothelium-independent relaxation) while the vein graft was no vascular reactivity because of endothelial injury but conserved a low relaxation mediated by SMC for high dose of sodium nitroprusside.

### Focus of the model

Models of venous grafting in the arterial position have been used to improve the patency of grafts in humans [35, 36], which is especially relevant in CABG. Goldman et al. [37] reported saphenous vein graft patency of 60% or more at 10 years postoperatively in humans. Animal models are useful to analyze the pathology of vein graft disease and to test therapeutic strategies in vivo [38]. In large animals such as pigs and dogs, the model consists of an interposition of saphenous and jugular veins into common carotid artery. Wan et al. reported rate patency at 4 weeks [39] with saphenous vein-common carotid artery interposition model in large white swine. In rats, different models of inferior vena cava into abdominal aorta interposition have been described as superficial epigastria vein into femoral artery interposition or ileolumbar vein into the abdominal aorta interposition [28]. In the surgical model proposed by Sun et al., the vein graft was interposed into the recipient’s abdominal aorta using microsurgical techniques in a latero-terminal configuration, and the aorta of the recipients was then ligated between the two anastomoses [28] while we developed an end-to-end anastomosis technique aligning vein and arterial endothelial cells in direction of arterial blood flow, therefore minimizing the effects of shear stress. Vein graft failure is characterized by an inflammatory response with leukocytes recruitment from circulating blood cells as we did not observe on the aorta segments. Like saphenous vein graft during CABG, our vein graft was submitted to a systolic flow. The vena cava was harvested using state-of-the-art and optimal handling techniques (same surgeon, a traumatic surgical technique, avoiding excessive handling and distortion) in order to reduce traumatic damage to the endothelium layer, which is the first step of the graft failure. The main difficulty in vein graft transplantation was the vein wall’s thickness. In fact, the texture of the vein issued from its storage solution makes the wall extremely fine.

### Limitations of the study

The high number of occluded vein grafts reduced the possibilities of analysis. Moreover, intermediate analysis of the vein grafts should have been performed in order to explain the mechanisms of vascular inflammation in relation of each group. Due to the small number of vein grafts sections we combined data from all storage solution groups in order to compare vascular reactivity to each solution.

## Conclusion

The composition of preservation solutions influences vein graft endothelial structural and functional integrity from early graft failure. However, the next crucial step will be conducted randomized clinical trial to compare storage solutions and long-term clinical outcomes because our experimental study show that some solutions do not protect the vein graft from intimal hyperplasia and thrombosis formation during the arterialization stage in this experimental study.

## Data Availability

The datasets used and/or analyzed during this study available from the corresponding author on reasonable request.

## Abbreviations

CABG: Coronary artery bypass grafting
CS: Cyclic strain
EC: Endothelial cells
ECM: Extracellular matrix
HW: Hydrogen-rich water
IH: Intimal hyperplasia
NOS: Nitric oxide synthetase
SMC: Smooth muscle cells
SS: Shear stress
SVG: Saphenous vein graft
